# Guard-Cell-Specific Expression of Phototropin2 C-Terminal Fragment Enhances Leaf Transpiration

**DOI:** 10.3390/plants11010065

**Published:** 2021-12-26

**Authors:** Young-Sun Riu, Hyun-Geun Song, Hwi-Su Kim, Sam-Geun Kong

**Affiliations:** 1 Department of Biological Sciences, College of Natural Sciences, Kongju National University, Chungnam 32588, Korea; youngsun432@smail.kongju.ac.kr (Y.-S.R.); 201301237@smail.kongju.ac.kr (H.-G.S.); gacv1726@smail.kongju.ac.kr (H.-S.K.); 2 Biotechnology Research Institute, Kongju National University, Chungnam 32588, Korea

**Keywords:** *Arabidopsis thaliana*, phototropin2, BLUS1 (BLUE LIGHT SIGNALING1), stomatal opening, transpiration

## Abstract

Phototropins (phot1 and phot2) are plant-specific blue light receptors that mediate chloroplast movement, stomatal opening, and phototropism. Phototropin is composed of the N-terminus LOV1 and LOV2 domains and the C-terminus Ser/Thr kinase domain. In previous studies, 35-P2CG transgenic plants expressing the phot2 C-terminal fragment–GFP fusion protein (P2CG) under the control of *35S* promoter showed constitutive phot2 responses, including chloroplast avoidance response, stomatal opening, and reduced hypocotyl phototropism regardless of blue light, and some detrimental growth phenotypes. In this study, to exclude the detrimental growth phenotypes caused by the ectopic expression of P2C and to improve leaf transpiration, we used the *PHOT2* promoter for the endogenous expression of GFP-fused P2C (GP2C) (P2-GP2C) and the *BLUS1* promoter for the guard-cell-specific expression of GP2C (B1-GP2C), respectively. In P2-GP2C plants, GP2C expression induced constitutive phototropin responses and a relatively dwarf phenotype as in 35-P2CG plants. In contrast, B1-GP2C plants showed the guard-cell-specific P2C expression that induced constitutive stomatal opening with normal phototropism, chloroplast movement, and growth phenotype. Interestingly, leaf transpiration was significantly improved in B1-GP2C plants compared to that in P2-GP2C plants and WT. Taken together, this transgenic approach could be applied to improve leaf transpiration in indoor plants.

## 1. Introduction

Plants, as sessile organisms, have evolved sophisticated photoreceptor systems to adapt to fluctuating light environments. The photoreceptors include ultraviolet-B (UV-B)-absorbing UVR8, UV-A/blue light-absorbing cryptochromes, phototropins, members of the Zeitlupe family, and red/far-red light-absorbing phytochromes [1]. Of those, phototropins (phot1 and phot2 in Arabidopsis) mediate a variety of blue-light responses, including chloroplast photorelocation movement, phototropism, stomatal opening, and leaf-flattening, all of which are essential to optimize the photosynthetic ability of plants [2,3,4,5,6,7,8].

Chloroplasts change their intracellular location according to the intensity and position of incident light [9]. Under weak light conditions, chloroplasts accumulate to the periclinal sides of the mesophyll cells to maximize light absorption (accumulation response). In contrast, under strong light conditions, chloroplasts move to the anticlinal sides of the mesophyll cells to minimize light absorption (avoidance response). Therefore, the accumulation response is necessary to optimize photosynthetic ability and plant growth in Arabidopsis [10], and the avoidance response is essential for plant survival under excess light conditions [4,5]. In addition, chloroplasts accumulate at the bottom of the mesophyll cells in the dark (dark response) [11].

In Arabidopsis, chloroplast movement is regulated by the blue-light photoreceptor phototropins, phot1 and phot2. The accumulation response is mediated redundantly by both phot1 and phot2, while the avoidance response and dark response are mediated solely by phot2 [4,6,11,12]. Both phot1 and phot2 also redundantly mediate the blue-light-induced phototropic response that is induced by asymmetric auxin distribution patterns according to the light direction [13,14]. In Arabidopsis, phot1 mediates hypocotyl phototropism at both low-intensity (0.01 to 1 µmol m^−2^ s^−1^) and high-intensity (>1 µmol m^−2^ s^−1^) blue light (BL), whereas phot2 functions only at high-intensity BL [6]. The stomatal opening is also redundantly regulated by phot1 and phot2 [15,16,17]. Phototropin is autophosphorylated by BL, which activates the plasma membrane H⁺-ATPase to induce H⁺ export and sequentially K⁺-channel to induce influx of K⁺ and osmotic pressure [17,18,19]. BLUE LIGHT SIGNALING1 (BLUS1), as a phototropin kinase substrate, is specifically involved in stomatal opening by regulating signaling components, including type 1 protein phosphatase (PP1), PP1 regulatory subunit2-like protein1 (PRSL1), and 14-3-3 protein [20,21,22]. BLUS1 is specifically expressed in guard cells [23].

Phototropins comprise two light-oxygen-voltage (designated LOV1 and LOV2) domains at the N-terminus and Ser/Thr kinase domain at the C-terminus [15]. The LOV domains function as versatile photosensory modules, which are highly conserved in plants as well as other organisms including bacteria, archaea, and fungi [24]. The LOV domains absorb a broad range of wavelengths from UV-A to blue light (350–500 nm) through the flavin mononucleotide (FMN) chromophore, which photochemically forms a covalent bond with the conserved Cys residue in the LOV domains [25,26]. In particular, the LOV2 domain plays the essential role of regulating the activity of the kinase in a BL-dependent manner via the conformational changes between the LOV2 domain and its linked C-terminal Jα helix [27,28,29]. During light activation, the BL-induced autophosphorylation of phototropins is the primary step for downstream signaling [16,23,30,31,32].

Most of phot1 and phot2 are localized to the plasma membrane in the dark. Some fractions of phot1 are released from the plasma membrane to the cytosolic fractions, and some fractions of phot2 are associated with the Golgi apparatus in response to BL [33]. Some fractions of phot1 and phot2 are also associated with the outer membrane of chloroplast [34]. Different subcellular localizations of phot2 represent the differential cellular functions [35]. The plasma membrane localization of phototropin is mediated by the C-terminal kinase domain [36,37]. The transgenic plants expressing the phot2 C-terminal fragment (P2C)–green fluorescent protein (GFP) fusion gene under the control of *35S* promoter (35-P2CG) show constitutive avoidance response and stomatal opening regardless of BL. In addition, 35-P2CG plants also exhibit reduced hypocotyl phototropism and some detrimental growth phenotypes such as dwarfism and infertility [36].

In this study, we aimed to overcome the detrimental effects caused by the ectopic expression of P2C under the control of *35S* promoter in 35-P2CG. The *PHOT2* promoter [33] and the *BLUS1* promoter [23] were used to express P2C in an endogenous-like and a guard-cell-specific manner in P2-GP2C and B1-GP2C plants. P2-GP2C plants showed the constitutive phot2 responses with an alleviated vegetative growth phenotype. In contrast, B1-GP2C plants showed a normal vegetative growth phenotype, chloroplast photorelocation movement, phototropism, and hypocotyl growth. Interestingly, B1-GP2C plants exhibited constitutive stomatal opening regardless of BL and enhanced leaf transpiration.

## 2. Results

### 2.1. B1-GP2C Plants Express P2C in a Guard-Cell-Specific Manner

**Figure 1:**
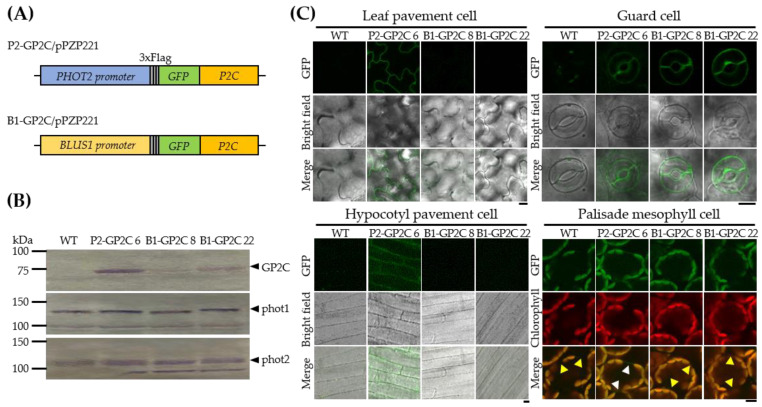
Production of P2-GP2C and B1-GP2C transgenic Arabidopsis plants. (**A**) Schematic diagrams of the P2-GP2C and B1-GP2C expression vectors used for *Agrobacterium*-mediated Arabidopsis transformation. The detailed cloning procedures are discussed in the Material and Methods section. (**B**) Immunoblot analysis of P2-GP2C and B1-GP2C plants. Total proteins were extracted from the rosette leaves of 3-week-old WT, P2-GP2C, and B1-GP2C plants. The blots were probed with anti-Flag (**top**), anti-phot1 (**middle**), and anti-phot2 (**bottom**) antibodies, respectively. (**C**) Tissue-specific expression patterns of GP2C in P2-GP2C and B1-GP2C plants. GFP fluorescence was observed by confocal microscopy. Note that the fluorescent signals for GFP obtained from palisade mesophyll cells also contained autofluorescent signals from chlorophyll. GFP fluorescence was confirmed in the plasma membrane of mesophyll cells in P2-GP2C plant (white arrow) but not in WT and B1-GP2C plants (yellow arrow). Scale bar = 10 μm.

To exclude the detrimental effects caused by the ectopic expression of P2C [36], we expressed P2C in an endogenous-like and a guard-cell-specific manner. To express P2C in a guard-cell-specific manner, P2C was fused with 3xFlag tag and GFP genes (GP2C), and the fusion gene was expressed under the control of the native *BLUS1* promoter in Arabidopsis transgenic plants (B1-GP2C) (Figure 1A). The native *PHOT2* promoter was also used as the constitutive expression control of GP2C (P2-GP2C) (Figure 1A). The T3 homogeneous lines were used for all the following analyses. The expression levels of GP2C in the leaf tissues of three-week-old transgenic plants were examined by Western blot analysis using an anti-Flag antibody (Figure 1B). The expression levels of GP2C in P2-GP2C plants were higher than those in B1-GP2C plants. The expression levels of the endogenous phot1 and phot2 in the transgenic plants were comparable to those of the wild-type (WT), indicating that GP2C expressions did not affect the endogenous phot1 and phot2 expressions.

We next examined the tissue-specific expression patterns and subcellular localizations of GP2C in P2-GP2C and B1-GP2C plants. The leaf tissues of three-week-old transgenic plants were subjected to confocal microscopy to observe the subcellular localization of GP2C (Figure 1C). The GFP fluorescent signals in P2-GP2C plants were detected in the plasma membrane of the pavement cells, the palisade mesophyll cells, and the guard cells, while the GFP fluorescent signals in B1-GP2C plants were only detected in the plasma membrane of the guard cells. Hence, it was confirmed that GP2C in B1-GP2C plants was specifically expressed in the guard cell under the control of the *BLUS1* promoter. 

### 2.2. B1-GP2C Plants Show Normal Chloroplast Photorelocation Movement

**Figure 2:**
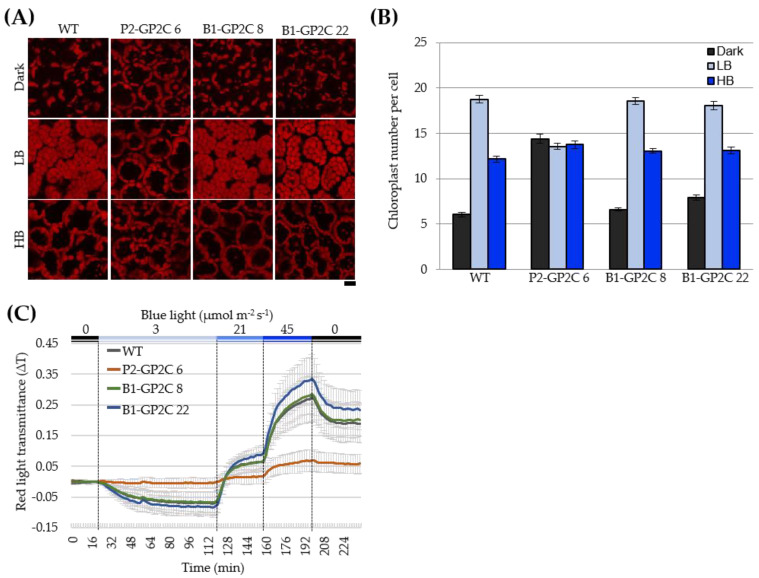
Chloroplast photorelocation movement in WT, P2-GP2C, and B1-GP2C plants. (**A**) Chloroplast positioning in different light conditions. The third and fourth rosette leaves were detached from 3-week-old Arabidopsis plants and kept on agar plates containing 0.8% gellan gum in the dark for 14 h or under low intensity of blue light (LB, 2 μmol m^−2^ s^−1^) for 3 h or high intensity of blue light (HB, 50 μmol m^−2^ s^−1^) for 2 h. The rosette leaves were kept in the fixation buffer before observation. Chloroplast positioning was observed using confocal laser scanning microscopy. Scale bar = 10 μm. (**B**) Chloroplast number in the palisade mesophyll cells of rosette leaves of WT, P2-GP2C, and B1-GP2C plants shown in (**A**). Data represent the mean ± SE (*n* = 10 cells). (**C**) Changes in the red-light (RL) transmittance were traced in the third and fourth rosette leaves of 3-week-old Arabidopsis plants under different light conditions. The rosette leaves were sequentially irradiated with blue light at different intensities of 0 μmol m^−2^ s^−1^ for 20 min, 3 μmol m^−2^ s^−1^ for 100 min, 21 μmol m^−2^ s^−1^ for 40 min, 45 μmol m^−2^ s^−1^ for 40 min, and 0 μmol m^−2^ s^−1^ for 40 min. Data represent the mean ± SE of three independent experiments (*n* = 3).

In order to address whether GP2C is constitutively active in phot2 responses or not in P2-GP2C and B1-GP2C plants as in 35-P2CG plants [36], we first observed chloroplast positioning under various light conditions using WT as the control. When WT and B1-GP2C plants were incubated in the dark for 14 h, chloroplasts were sparsely positioned in the periclinal surface of the palisade mesophyll cells of rosette leaves (Figure 2A). When the rosette leaves were incubated in a low intensity of BL (LB, 2 μmol m^−2^ s^−1^) for 2 h, chloroplasts were intensively observed in the periclinal surface of the palisade mesophyll cells of WT and B1-GP2C plants (Figure 2A). Consistently, the number of chloroplasts in the periclinal surface of the palisade mesophyll cells of WT and B1-GP2C plants was increased compared to those in the dark (Figure 2B). When the rosette leaves were exposed to a high intensity of BL (HB, 50 μmol m^−2^ s^−1^), the chloroplasts were observed in the anticlinal sides of the palisade mesophyll cells of WT and B1-GP2C plants due to the avoidance response (Figure 2A). The number of chloroplasts in the periclinal surface of the palisade mesophyll cells of WT and B1-GP2C plants was similarly reduced under HB compared to those under LB (Figure 2B). However, in P2-GP2C plants, the chloroplasts in the palisade mesophyll cells were constantly observed at the anticlinal sides with similar chloroplast numbers regardless of the light conditions (Figure 2A,B).

Chloroplast photorelocation movement was also analyzed through the changes of red light (RL) transmittance of rosette leaves [38]. In WT and B1-GP2C plants, the treatment of LB (3 μmol m^−2^ s^−1^) induced a decrease in RL transmittance due to the chloroplast accumulation response (Figure 2C). On the other hand, the treatments of HB (21, 45 μmol m^−2^ s^−1^) gradually increased RL transmittances due to the chloroplast avoidance response. When BL was turned off, RL transmittances were decreased due to the chloroplast dark reaction (Figure 2C). In P2-GP2C plants, RL transmittance was not significantly changed at all the light intensities due to the constitutive chloroplast avoidance response (Figure 2A–C). Taken together, B1-GP2C plants showed normal chloroplast photorelocation movement, although P2-GP2C plants showed a constitutive chloroplast avoidance response.

### 2.3. B1-GP2C Plants Show Normal Hypocotyl Growth and Phototropic Response

**Figure 3:**
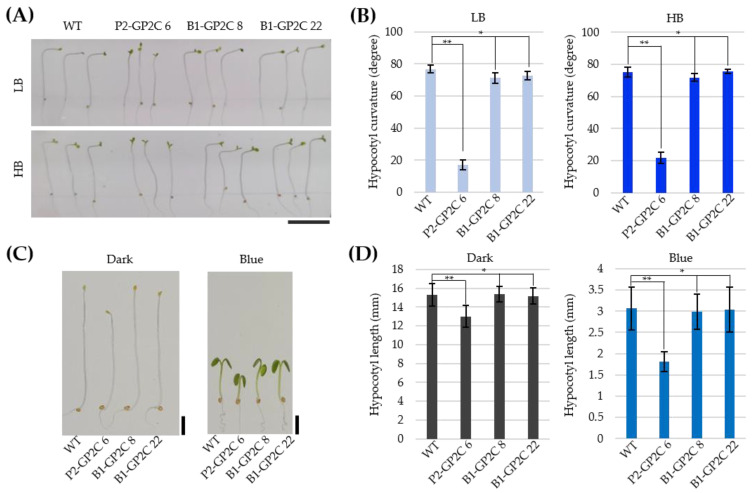
Hypocotyl growth and phototropic responses in WT, P2-GP2C, and B1-GP2C plants. (**A**) Photos showing the phototropic phenotypes of WT, P2-GP2C, and B1-GP2C plants. Three-day-old dark grown Arabidopsis seedlings were irradiated with low intensity of blue light (LB, 2 μmol m^−2^ s^−1^) and high intensity of blue light (HB, 40 μmol m^−2^ s^−1^) for 16 h. Scale bar = 1 cm. (**B**) Phototropic curvature was measured as the change in hypocotyl angle as determined from an analysis of stacked images of (**A**). Data represent the mean ± SE (*n* = 15). (**C**) WT, P2-GP2C, and B1-GP2C seedlings were grown in the dark or blue light (10 μmol m^−2^ s^−1^) for 5 days. Scale bars = 2 mm. (**D**) Quantitative analyses of the hypocotyl length of seedlings shown in (**C**). Data represent the mean ± SE of three independent experiments (*n* > 25). Asterisks indicate statistical differences detected by Student’s *t*-test (* not significant *p* > 0.05; ** significant *p* < 0.0001).

In previous studies, 35-P2CG plants exhibited reduced hypocotyl phototropism and elongation due to constitutive P2C activity [36]. Therefore, we first examined the hypocotyl phototropism of P2-GP2C and B1-GP2C plants in response to LB (2 μmol m^−2^ s^−1^) and HB (40 μmol m^−2^ s^−1^) (Figure 3A,B). P2-GP2C plants exhibited reduced phototropic responses at both LB and HB conditions as in 35-P2CG plants [36]. In contrast, the hypocotyl phototropic response was normally observed in B1-GP2C plants as in WT (Figure 3A,B). We next examined hypocotyl lengths. The hypocotyl lengths of P2-GP2C seedlings were significantly shorter than those of WT in the dark as well as at 10 μmol m^−2^ s^−1^ of BL. However, the hypocotyl lengths of B1-GP2C seedlings were similar to those of WT (Figure 3C,D). It was concluded that B1-GP2C transgenic lines were normal in hypocotyl growth and phototropic response unlike P2-GP2C transgenic lines showing reduced hypocotyl growth and phototropic response.

### 2.4. B1-GP2C Plants Show Normal Growth Phenotypes

**Figure 4:**
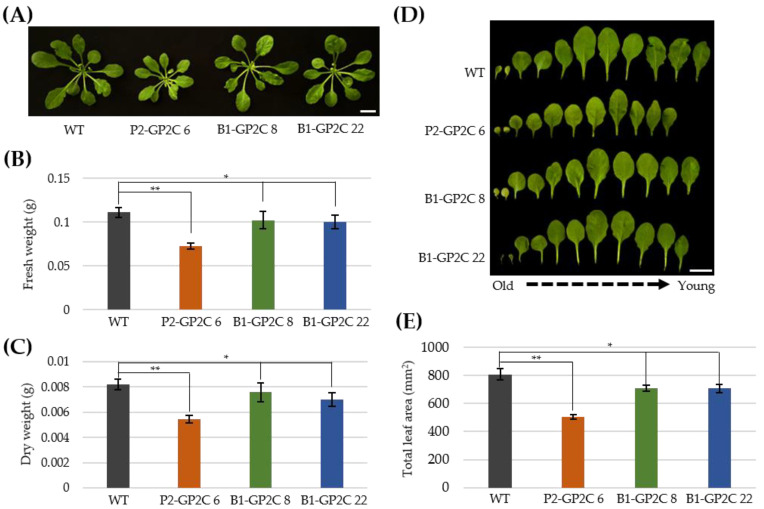
Vegetative growth phenotypes in P2-GP2C and B1-GP2C plants. (**A**–**E**) Comparisons of the rosette leaf growth (**A**), fresh weight (**B**), dry weight (**C**), leaf growth (**D**), and total leaf area (**E**) of 6-week-old WT, P2-GP2C, and B1-GP2C plants. Scale bars = 1 cm. Data represent the mean ± SE (*n* = 15 in B and C, *n* = 10 in E). Asterisks indicate statistical differences detected by Student’s *t*-test (* not significant *p* > 0.05; ** significant *p* < 0.0001).

Ectopic expression of P2C in 35-P2CG plants induced some detrimental effects on vegetative and reproductive plant growth, including a reduction in rosette leaf size, shoot apical dominance, and male sterility [36]. Although P2-GP2C plants exhibited some dwarf phenotypes with smaller rosette leaves compared to those of WT and B1-GP2C plants, seed setting was normally observed (Figure 4A). Consistently, the fresh weight and dry weight of P2-GP2C plants were approximately 65.4% and 66.6% of those of WT, respectively (Figure 4B,C). The fresh weight and dry weight of B1-GP2C plants were approximately 90.1–92.1% and 85.7–92.4% of those of WT, respectively, although the difference between the WT and B1-GP2C plants was not statistically significant (Figure 4B,C). In addition, the whole leaf area of the P2-GP2C plant was approximately 60.2% of that of WT. In contrast, the whole leaf area of B1-GP2C plants was 79–84% of that of WT, a difference that was statistically not significant (Figure 4D,E). Hence, it was concluded that B1-GP2C plants were normal in plant growth, while P2-GP2C plants showed some alleviated detrimental growth phenotype with normal fertility, rather than 35-P2CG plants [36].

### 2.5. Both P2-GP2C and B1-GP2C Plants Show Constitutive Stomatal Opening Regardless of Blue Light

**Figure 5:**
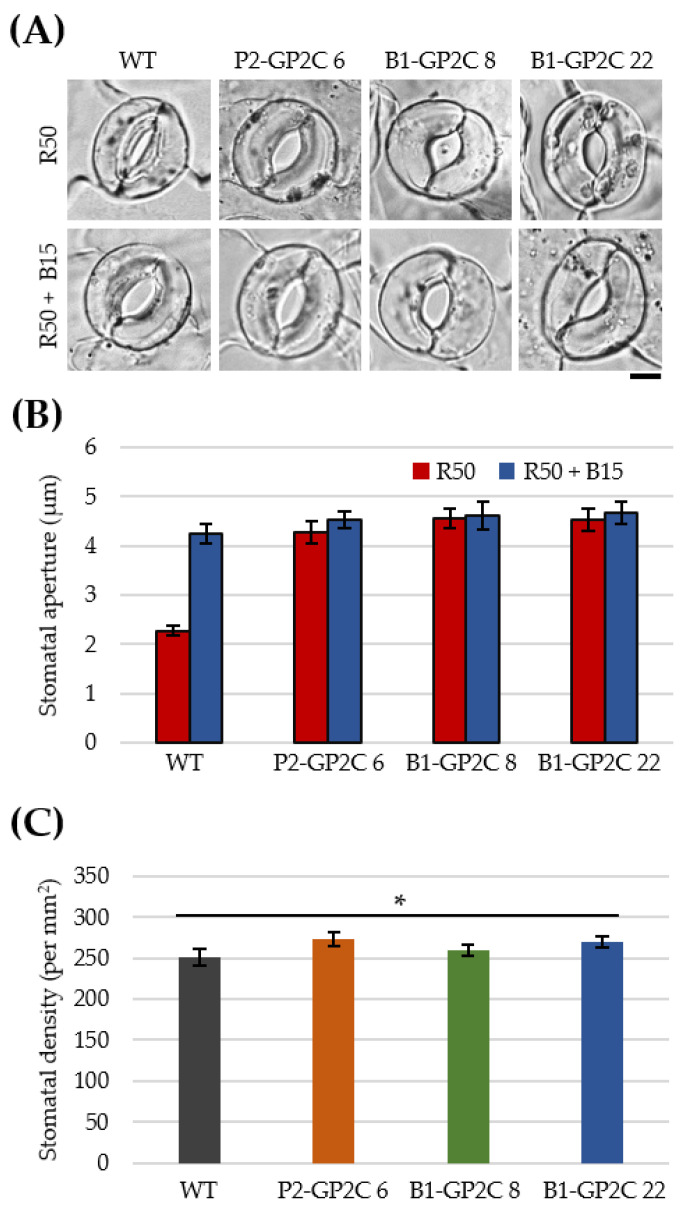
Stomatal apertures in P2-GP2C and B1-GP2C plants. (**A**) Confocal images showing the stomatal opening of the WT, P2-GP2C, and B1-GP2C plants. Epidermal strips were detached and preincubated for 1 h in darkness and then illuminated by red light (R50, 50 μmol m^−2^ s^−1^) with or without blue light (B15, 15 μmol m^−2^ s^−1^) for 3 h. Scale bar = 5 μm. (**B**) Stomatal apertures in the WT, P2-GP2C, and B1-GP2C plants under the same conditions as A. Data represent the mean ± SE (*n* = 20). (**C**) Stomatal density of 4-week-old plants. Data represent the mean ± SE (*n* = 10 independent leaves). Asterisk indicates statistical difference detected by Student’s *t* test (* not significant *p* > 0.05).

We next examined whether GP2C had constitutive activity on stomatal opening. To examine stomatal opening, we measured stomatal apertures at 50 μmol m^−2^ s^−1^ of RL (R50) and R50 superimposed with 15 μmol m^−2^ s^−1^ of BL (R50 + B15) (Figure 5A,B). The stomatal apertures in WT were wider under R50 + B15 compared with those under R50. By contrast, stomata in P2-GP2C and B1-GP2C plants were opened regardless of the light conditions (Figure 5A,B). Hence, it was concluded that GP2C in B1-GP2C and P2-GP2C plants activated a full stomatal response regardless of BL as previously described in 35-P2CG plants [36]. In addition, the shapes and densities of stomata in the epidermis of the rosette leaves were similarly observed in WT and the transgenic plants (Figure 5A,C).

### 2.6. B1-GP2C Plants Show Enhanced Transpiration

**Figure 6:**
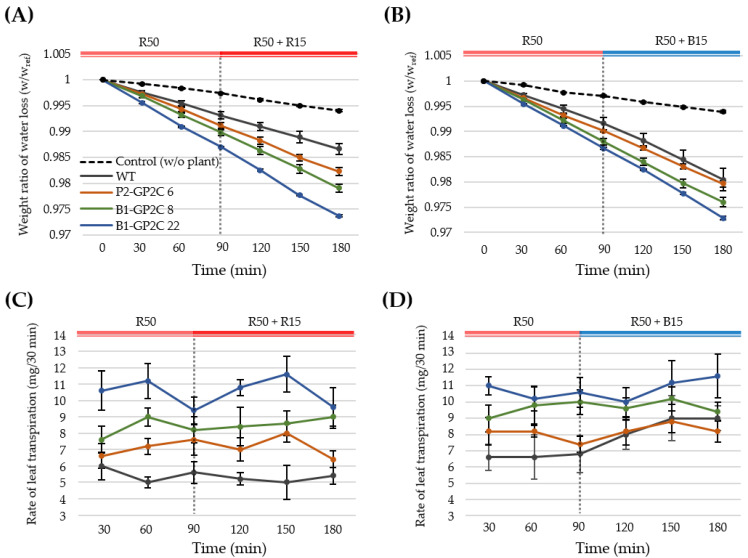
Leaf transpirations in P2-GP2C and B1-GP2C plants. (**A**) Temporal changes in weight loss by leaf transpiration measured at 30 min intervals for 90 min under red light (R50, 50 μmol m^−2^ s^−1^) and for an additional 90 min under red light (15 μmol m^−2^ s^−1^) superimposed on red light (R50 + R15, 50 μmol m^−2^ s^−1^). (**B**) Temporal changes in weight loss by leaf transpiration measured at 30 min intervals for 90 min under red light (R50, 50 μmol m^−2^ s^−1^) and for an additional 90 min under blue light (15 μmol m^−2^ s^−1^) superimposed on red light (R50 + B15, 50 μmol m^−2^ s^−1^). Weight loss ratios were obtained from five plants at each time point. (**C**) Actual water reduction in the condition of (**A**). (**D**) Actual water reduction in the condition of (**B**). Data represent the mean ± SE (*n* = 5).

Since stomatal opening is necessary to induce leaf transpiration, we next examined leaf transpiration of P2-GP2C and B1-GP2C plants under sequential light treatment with 50 μmol m^−2^ s^−1^ of RL (R50) as background light superimposed with 15 μmol m^−2^ s^−1^ of RL (R50 + R15) or BL (R50 + B15) (Figure 6). Microbalance was used to measure the changes of water loss due to leaf transpiration at 30 min intervals. The weights were calculated from the measured weight divided by the initial weight (W/W_ref_) and statistically averaged (Figure 6A,B). In the condition of R50, the water loss in WT was faster than the natural transpiration in the opened E-tube used as a negative control. However, the water loss in WT was less than those in P2-GP2C and B1-GP2C plants under the condition of R50. Interestingly, B1-GP2C plants exhibited a higher water loss compared to that in P2-GP2C plants (Figure 6A). The results were similarly observed at the condition of the RL superimposed with BL (R50 + R15) (Figure 6A). In the condition of RL superimposed with BL (R50 + B15), the water loss in WT was slightly increased compared to that in the light conditions of RL only (R50 and R50 + R15) (Figure 6B). Nevertheless, under the condition of R50 + B15, the water loss in WT was less than that of B1-GP2C plants, although the water loss in WT was comparable to that of the P2-GP2C plant (Figure 6B).

We further analyzed the rate of leaf transpiration by calculating the rate of water loss per 30 min (Figure 6C,D). Interestingly, B1-GP2C plants exhibited the higher rate of leaf transpiration compared to that in not only WT but also in P2-GP2C plants in the condition of RL (R50 and R50 + R15) (Figure 6C). In contrast, leaf transpiration of the WT was gradually increased and finally comparable to that of the P2-GP2C plant after BL treatment for 90 min (R50 + B15) (Figure 6D). Hence, leaf transpirations in B1-GP2C and P2-GP2C plants were constitutively higher than that in WT regardless of light conditions (Figure 6D). The increase in leaf transpiration in B1-GP2C and P2-GP2C plants is well-consistent with their phenotypic trait on constitutive stomatal opening.

## 3. Discussion

In this study, we produced transgenic Arabidopsis plants expressing GFP-phot2 C-terminus (GP2C) under the controls of the *PHOT2* promoter and the *BLUS1* promoter in an endogenous-like and a guard-cell-specific manner, respectively (Figure 1A,B). GP2C expression in P2-GP2C plants induced constitutive phot2 responses on phototropism, stomatal opening, and chloroplast movement regardless of BL as previously described in 35-P2CG [36]. However, P2-GP2C plants showed some detrimental growth phenotypes in hypocotyl growth and vegetative growth (Figure 3 and Figure 4). In contrast, GP2C expression in B1-GP2C plants induced only constitutive stomatal opening with normal phototropism, chloroplast movement, and vegetative growth (Figure 2, Figure 3, Figure 4 and Figure 5). GP2C was expressed in the plasma membrane of various tissues, including leaf and hypocotyl tissue in P2-GP2C plants (Figure 1C). In contrast, GP2C was specifically expressed in the guard cells in B1-GP2C plants (Figure 1C).

phot2 functions in the hypocotyl phototropic response, chloroplast movement, and stomatal opening. The native *PHOT2* promoter has light-inducible and universal expression activities, by which the expression of phot2-GFP (P2G) fully complements the *phot2*-deficient mutant phenotypes [33]. In addition, the subcellular localization of GP2C is well-consistent with the plasma membrane localization of P2G via the C-terminus (Figure 1C; [33,36,37]). BLUS1 is specifically expressed in guard cells and functions only in the stomatal opening [23]. Depending on the tissue-specific expression patterns of the native *PHOT2* and *BLUS1* promoters, B1-GP2C and P2-GP2C plants exhibited distinct phenotypic characteristics. A dwarf phenotype was observed in P2-GP2C plants but not in B1-GP2C plants (Figure 4). In previous studies, 35-P2CG plants showed a dwarf phenotype with a reduction in rosette leaf size, shoot apical dominance, and male sterility [36]. Most of these characteristics were consistently observed by ectopic expressions of phot2 or P2C under the control of the *35S* promoter [33,36,37]. On the other hand, P2-GP2C plants showed an alleviated phenotype on seed set that was normally produced by self-pollination, indicating that there are different promoter activities between the *35S* promoter and the *PHOT2* promoter [33,36,39]. Unlike the two transformants, B1-GP2C plants showed a normal growth phenotype similarly to the WT (Figure 4). These results might be due to the guard-cell-specific expression of P2C under the control of the *BLUS1* promoter (Figure 1C).

In previous studies, 35-P2CG plants exhibited constitutive stomatal opening regardless of BL [36]. Likewise, constitutive stomatal opening was observed in both P2-GP2C and B1-GP2C plants (Figure 5). The activity of phototropin kinase acts as an initial signal of stomatal opening by phosphorylating BLUS1 [23]. These initial signals mediate stomatal opening by regulating the phosphorylation of H^+^-ATPase and activation through the pathways of signaling factors located in the guard cell membrane [17,20,21,23]. Thus, P2C constitutively activates a series of the signal transduction pathway to induce stomatal opening.

Chloroplasts change intracellular localizations according to light intensity and position [9]. The avoidance response is only mediated by phot2 [4,6,12]. In accordance with the previous results of 35-P2CG [36], chloroplasts in P2-GP2C plants exhibit avoidance response even in the dark, indicating that P2C is constitutively active on chloroplast movement regardless of light (Figure 2). However, chloroplast photorelocation movements in B1-GP2C plants were normally observed as WT (Figure 2). These data suggest that GP2C expressed in the guard cell by the *BLUS1* promoter only functions in the stomatal opening but not in other phot2 responses, including chloroplast movement and phototropism, further suggesting that phot2 is functional cell-autonomously. Consistently, phot2 functions cell-autonomously in chloroplast movement [4].

Phototropin is autophosphorylated in response to BL. The autophosphorylation of phototropin is the initial signaling step to activate phototropin kinase [16,32]. Phototropin triggers the downstream signaling by phosphorylating protein factors such as the ATP-BINDING CASSETTE B19 (ABCB19) and PIN-FORMED (PIN) protein families of auxin transporters that are involved in asymmetric auxin distribution [40,41] and the BLUS1 in stomatal opening [23]. Such asymmetric distribution of auxin induces phototropism. The ectopic expression of P2CG in the 35-P2CG plant interfered with the asymmetric distribution of auxin in hypocotyl, thereby inducing high levels of expression of the DR5::GUS reporter gene and reduced hypocotyl elongation and phototropism [36]. Since the expression of GP2C was observed in the hypocotyl (Figure 1C), it is plausible that the constitutive activity of P2C in the hypocotyl could interfere with the asymmetric distribution of auxin, as in the 35-P2CG plants (Figure 3A,B). In contrast, B1-GP2C plants showed no expression of GP2C in hypocotyl (Figure 1C). Therefore, hypocotyl growth and phototropism were normally observed in the B1-GP2C plants, as in WT (Figure 3A,B). Consistently, hypocotyl growth in the P2-GP2C plants was shorter than those of WT and B1-GP2C plants (Figure 3C,D). Taken together, we conclude these data result from the tissue-specific expressions of P2C.

The B1-GP2C plants exhibited constitutive stomatal opening; as a result, leaf transpiration was effectively increased. Since the stomatal opening increases the amount of transpiration [17,42], the water loss by leaf transpiration in P2-GP2C and B1-GP2C plants was increased compared to that in WT due to the constitutive stomatal opening (Figure 6). Among these, the B1-GP2C plants had a faster transpiration rate than the P2-GP2C plants. The rate of transpiration is mainly regulated by stomatal movement, but in many plants, it is also regulated by stomata density and number [43,44,45]. However, since there was no difference in stomata density between the two transformants (Figure 5C), the transpiration rate was different depending on the total number of stomata due to the difference in leaf area (Figure 4E). In addition, the transpiration rate increases as the weight of the aboveground part of the plant increases [46,47,48]. P2-GP2C plants showed a smaller leaf area due to their dwarfism, resulting in leaf transpiration lower than that of B1-GP2C plants. Therefore, the guard-cell-specific expression of P2C in B1-GP2C plants effectively induced constitutive stomatal opening, thus increasing the rate of transpiration.

Plants not only have excellent efficacy in removing air pollutants from indoor environments but also act as eco-friendly humidifiers by discharging moisture [49,50,51]. The ability to purify these pollutants and release moisture occurs through the stomata [52,53]. Therefore, transgenic plants with a constitutive stomatal opening are thought to have better air purification ability, and these physiological characteristics could be also applied to plants with excellent air purification ability. This approach would be challenged as a subject of future study.

## 4. Materials and Methods

### 4.1. Plant Materials and Growth Conditions

All *Arabidopsis thaliana* lines used in this study were of *gl-1* (ecotype Columbia) background. To analyze the plant phenotype, the seeds of WT and transgenic plants (see below) were surface-sterilized with a sterilization solution containing 4% sodium hypochlorite (NaOCl) and 0.1% Triton X-100 and sown on half-strength Murashige and Skoog (MS) medium supplemented with 1% (*w/v*) sucrose and 0.8% (*w/v*) plant agar (pH 5.8). The plants were grown in a growth chamber under white light (100 μmol m^−2^ s^−1^) with a 16/8 h light/dark cycle at 23 °C. For physiological analyses of stomatal opening, chloroplast movement, vegetative plant growth, and leaf transpiration, plants were grown on half-strength MS medium for 10 days in the growth chamber and were then transplanted onto vermiculite soil irrigated with mineral nutrients. The plants were further grown until use for appropriate physiological experiments under white light (100 μmol m^−2^ s^−1^) with a 16/8 h light/dark cycle at 23 °C and 50–60% humidity.

### 4.2. Binary Vector Construction and Agrobacterium-Mediated Transformation

The vectors for *Agrobacterium*-mediated *Arabidopsis* transformation were constructed by modifying the P2-P2G/pPZP221 vector [33,36]. For endogenous expression of P2C, the *PHOT2* promoter: 3x Flag fusion gene (P2-3FG) was constructed by PCR using the specific primer set (P2pro-Fw primer: 5′- TCAGCGGTCCTTTACCCGCCTC-3′ and P2Pro-3xFLAG_rv/*Sac*1 + *Sal*1 primer: 5′-AAGTCGACGAGCTCCTTGTCATCGTCATCC-3′) to produce a P2-3FG/pPZP221 vector. The P2C gene was cloned by PCR using the specific primer set (P2_1603fw/*Sal*1 primer: 5′-AAGTCGACATGGCGCGGCCCGAAGACCTG-3′ and P2_2745rv*/*Pst*1 primer: 5′-AACTGCAGTTAGAAGAGGTCAATGTCCA-AGTC-3′) and inserted into P2-3FG/pPZP221 using the restriction enzyme sites *Sal*I and *Pst*I to produce P2-GP2C/pPZP221 (Figure 1A).

To construct the B1-3FG/pPZP211 vector for guard-cell-specific expression of P2C, the *BLUS1* promoter was cloned from Arabidopsis genomic DNA by PCR using the specific primer set (BLUS1pro-Fw primer: 5′-GCTTTAGGAATGTTGAAAGTATTC-3’ and BLUS1pro-Rv primer: 5′-AAATAGAAAGAAGATGAGTACACTC-3′). The DNA fragments of the *BLUS1* promoter and 3FG were ligated into the *Hin*dIII and *Sal*I restriction enzyme sites of the pBluescript SK+ plasmid to produce B1-3FG/pBS. The B1-3FG/pPZP221 was prepared by substituting the P2-3FG region of P2-3FG/pPZP221 with the DNA fragment of B1-3FG prepared from B1-3FG/pBS using *Hin*dIII and *Sal*1 restriction sites. The P2C gene was inserted into B1-3FG/pPZP221 using the restriction enzyme sites *Sal*I and *Pst*I to produce B1-GP2C/pPZP221 (Figure 1A). The P2-GP2C/pPZP221 and B1-GP2C/pPZP221 vectors were used for WT Arabidopsis transformation using the *Agrobacterium*-mediated floral dip method [54]. Transformants were selected on the basis of antibiotic resistance against 35 μg/mL gentamicin, and independent homozygous T3 progenies were used for all experiments.

### 4.3. Protein Extraction and Western Blot Analysis

The expression levels of GP2C and phototropins (phot1 and phot2) in the WT and transgenic plants were determined by immunoblot analysis (Figure 1B). One hundred milligrams of rosette leaves were harvested from 3-week-old plants that were grown on half-strength MS medium and were immediately frozen using liquid nitrogen and stored at −80 °C before use. The frozen tissues were homogenized with a glass homogenizer in 300 µL extraction buffer (50 mM Tris-HCL, 150 mM NaCl, 1 mM MgCl_2_, 2 mM ethylene-diamine-tetraacetic acid (EDTA), 1 mM dithiothreitol (DTT), 1 mM phenylmethylsulfonyl fluoride (PMSF), and 10% Glycerol, at pH 7.5). The homogenized rosette leaves were centrifuged twice for 15 min at 8000× *g*, and the supernatant was used for immunoblot analysis. Protein concentration was measured by the Bradford assay using bovine serum albumin (BSA) as a reference protein. Immunoblot analysis was performed as previously described [33]. Fifty micrograms of total protein extract was separated by 7.5% SDS-polyacrylamide gel electrophoresis. The separated proteins were transferred onto a nitrocellulose blotting membrane (GE Healthcare, Freiburg, Germany) and probed with anti-phot1 and anti-phot2 polyclonal antibodies [34] and an anti-FLAG monoclonal antibody (Sigma-Aldrich, St. Louis, MO, USA).

### 4.4. Confocal Laser Scanning Microscopy and Image Analysis

GFP observation was carried out using a confocal microscope (SP5, Leica, Wetzlar, Germany) as previously described [34]. The third or fourth rosette leaves of 3-week-old plants were used for the observations of the pavement cell, guard cell, and mesophyll cell. Hypocotyl pavement cells were observed using Arabidopsis seedlings grown on half-strength MS medium in the dark for 5 days. The fluorescent signals were excited at 488 nm of multi-Ar laser and captured at the narrow-band regions of 500–550 nm for GFP and 610–700 nm for chlorophyll, respectively.

### 4.5. Chloroplast Photorelocation Movement

Chloroplast photorelocation movement and intracellular positioning were analyzed as previously described [38]. For the analysis of chloroplast photorelocation movement using RL transmittance, the third or fourth rosette leaves of 3-week old Arabidopsis plants were placed onto the wells of a 96-well plate containing 0.5% (*w/v*) gellan gum (Wako, Osaka, Japan), and the wells were covered with a transparent film with small holes to prevent moisture. The leaves were kept in the dark at 23 °C for 4 h before measurement. Thereafter, the leaves were sequentially treated with different intensities of blue light (0 μmol m^−2^ s^−1^, 3 μmol m^−2^ s^−1^, 21 μmol m^−2^ s^−1^, or 45 μmol m^−2^ s^−1^) for 2 min, and then RL transmittance was automatically recorded at 2 min intervals using a microplate reader (VersaMax; Molecular Devices, San Jose, CA, USA).

For analysis of chloroplast photorelocation movement using the microbeam irradiation of confocal microscopy [55], the third or fourth rosette leaves of 3-week-old Arabidopsis plants grown on 0.8% (*w/v*) agar plate containing half-strength MS medium were separately placed on a plate containing 0.5% (*w/v*) gellan gum. The leaves were treated in the dark for 16 h with low-intensity blue light (2 μmol m^−2^ s^−1^) or with high-intensity blue light (50 μmol m^−2^ s^−1^) for 30 min. Then, the leaves were immediately reacted in fixation solution (20 mM PIPES, 5 mM MgCl_2_, 0.5 mM PMSF, 1% (*w/v*) dimethyl sulfoxide, and 2.5% (*w/v*) glutaraldehyde) for 30 min [55].

### 4.6. Hypocotyl Growth and Phototropism

To observe the hypocotyl growth of Arabidopsis seedlings, surface sterilized seeds were plated on 0.8% (*w/v*) agar plate containing half-strength MS medium without sucrose. The plates were incubated in the dark at 4 °C for 3 days and irradiated with white light (50 μmol m^−2^ s^−1^) for 1 h to induce germination. Then, the seedlings were grown in the dark or continuous blue light (10 μmol m^−2^ s^−1^) for 5 days. Hypocotyl phototrophic response was analyzed by the method as previously described [36]. Seedlings were grown vertically on 0.8% (*w/v*) agar containing half-strength MS medium without sucrose in the dark for 3 days. After that, they were irradiated for 16 h with low-intensity blue light (2 μmol m^−2^ s^−1^) and high-intensity blue light (40 μmol m^−2^ s^−1^). The plates were photographed, and the hypocotyl lengths and internal hypocotyl angles for each seedling were measured using NIH ImageJ software (https://imagej.nih.gov/ij/).

### 4.7. Vegetative Growth Phenotype

Vegetative growth phenotypes of WT and transgenic plants were examined by fresh and dry weights and rosette leaf areas from 6-week-old Arabidopsis plants grown in the plant growth room. Fresh weight was measured using the aboveground part of plants excluding the roots. The leaf tissues were dried at 45 °C for seven days to measure the dry weight. Intact rosette leaves were fixed on a plastic flat using Scotch tape, and then photographed to obtain the projected leaf area. The leaf area was measured from the photographs using ImageJ software (https://imagej.nih.gov/ij/).

### 4.8. Stomatal Opening

Stomatal opening was analyzed as described previously [36]. All experiments were carried out between 08:00 and 14:00 in the dark with the aid of a dim red safelight. Five rosette leaves of 4-week-old Arabidopsis plants were harvested in a 45 mL Falcon tube containing 10 mL of MES/KOH buffer (10 mM MES-KOH (pH 6.15), 50 mM KCL, and 0.1 mM CaCl_2_) and homogenized with a homogenizer (Polytron PT 1200 Kinematica, Lucerne, Switzerland). The homogenized leaf tissues were collected by filtration using a 58 μm nylon mesh and were transferred to a 35 mm Petri dish containing 5 mL of MES/KOH buffer. Thereafter, the epidermal strips were incubated in the dark for 1 h and then irradiated with 50 μmol m^−2^ s^−1^ of RL (R50) or R50 superimposed with 15 μmol m^−2^ s^−1^ of BL (R50 + B15) for 3 h. Confocal images were obtained using confocal microscopy (SP5, Leica, Germany). The stomatal apertures were measured from the photographs using ImageJ software (https://imagej.nih.gov/ij/).

### 4.9. Leaf Transpiration

Leaf transpiration was measured from 08:00 in the morning in the dark with the aid of a dim red safelight. Four-week-old plants were set in a 4 mm hole in the cap of a 1.5 mL e-tube filled with 1.5 mL distilled water, in which only the aboveground part of the Arabidopsis plants came out from the tube and the root part was in the water of the e-tube. After dark treatment of Arabidopsis plants in the tube for 14 h, the tubes were weighed every 30 min under the following light conditions: 50 μmol m^−2^ s^−1^ of RL (R50) for 1 h 30 min and then R50 superimposed with 15 μmol m^−2^ s^−1^ of RL (R50 + R15) or 15 μmol m^−2^ s^−1^ of BL (R50 + B15) for 1 h 30 min, respectively.

### 4.10. Statistical Analysis

GraphPad Prism software (version 8.0.1) was used for statistical analysis. Student’s *t*-test was used to reveal any statistically significant differences between samples for the experiments.
